# Poly[(3-nitro­benzoato)(μ_3_-1,2,4-triazolato)cobalt(II)]

**DOI:** 10.1107/S1600536808043596

**Published:** 2009-01-08

**Authors:** Xu-Liang Qi

**Affiliations:** a Liaocheng Vocational and Technical College, LiaoCheng 252000, ShanDong, People’s Republic of China

## Abstract

In the title compound, [Co(C_2_H_2_N_3_)(C_7_H_4_NO_4_)]_*n*_, the Co^II^ atom is five-coordinated by three triazolate ligands and one bidentate 3-nitro­benzoate anion in a distorted trigonal-bipyramidal geometry. The triazolate ligand bridges the Co^II^ atoms, generating a two-dimensional net parallel to the *ab* plane, in which both the Co^II^ atom and the triazolate ligand act as three-connected nodes. Two weak inter­molecular C—H⋯O hydrogen bonds connect the nets.

## Related literature

For metal–triazole complexes, see: Park *et al.* (2006 ▶); Yang *et al.* (2008 ▶); Zhai *et al.* (2007 ▶). For Co—O and Co—N bond lengths, see: Zhang *et al.* (2008 ▶).
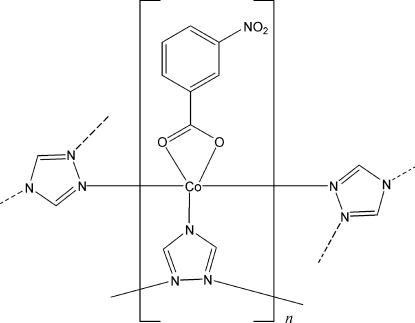


## Experimental

### 

#### Crystal data

[Co(C_2_H_2_N_3_)(C_7_H_4_NO_4_)]
*M* *_r_* = 293.11
Orthorhombic, 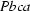

*a* = 9.2419 (18) Å
*b* = 10.377 (2) Å
*c* = 22.597 (5) Å
*V* = 2167.1 (8) Å^3^
*Z* = 8
Mo *K*α radiation
μ = 1.60 mm^−1^
*T* = 296 (2) K
0.14 × 0.12 × 0.12 mm

#### Data collection

Bruker SMART 1K CCD area-detector diffractometer
Absorption correction: multi-scan (**SADABS**; Sheldrick, 2004 ▶) *T*_min_ = 0.802, *T*_max_ = 0.826
19233 measured reflections
2477 independent reflections
2245 reflections with *I* > 2σ(*I*)
*R*_int_ = 0.029

#### Refinement

*R*[*F* ^2^ > 2σ(*F* ^2^)] = 0.023
*wR*(*F* ^2^) = 0.059
*S* = 1.04
2477 reflections
163 parameters
H-atom parameters constrained
Δρ_max_ = 0.33 e Å^−3^
Δρ_min_ = −0.28 e Å^−3^

### 

Data collection: *SMART* (Bruker, 2001 ▶); cell refinement: *SAINT* (Bruker, 2001 ▶); data reduction: *SAINT*; program(s) used to solve structure: *SHELXS97* (Sheldrick, 2008 ▶); program(s) used to refine structure: *SHELXL97* (Sheldrick, 2008 ▶); molecular graphics: *SHELXTL* (Sheldrick, 2008 ▶); software used to prepare material for publication: *SHELXTL*.

## Supplementary Material

Crystal structure: contains datablocks I, global. DOI: 10.1107/S1600536808043596/is2370sup1.cif

Structure factors: contains datablocks I. DOI: 10.1107/S1600536808043596/is2370Isup2.hkl

Additional supplementary materials:  crystallographic information; 3D view; checkCIF report

## Figures and Tables

**Table 1:** Selected bond lengths (Å)

Co1—O1	2.3314 (12)
Co1—O2	2.0008 (12)
Co1—N1	2.0232 (12)
Co1—N2^i^	2.0118 (12)
Co1—N3^ii^	2.0385 (12)

Symmetry codes: (i) 
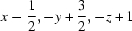
; (ii) 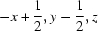.

**Table 2:** Hydrogen-bond geometry (Å, °)

*D*—H⋯*A*	*D*—H	H⋯*A*	*D*⋯*A*	*D*—H⋯*A*
C3—H3⋯O2^iii^	0.93	2.54	3.250 (3)	134
C8—H8⋯O4^iv^	0.93	2.46	3.372 (2)	169

Symmetry codes: (iii) 
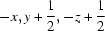
; (iv) 
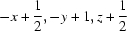
.

**Fig. 1.**
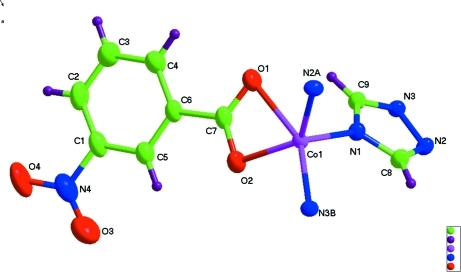
An ORTEP view of the asymmetric unit with 50% thermal ellipsoids for non-H atoms [symmetry codes: (A) -x + 1/2, y - 1/2, z; (B) x + 1/2, -y + 3/2, -z + 1].

**Fig. 2.**
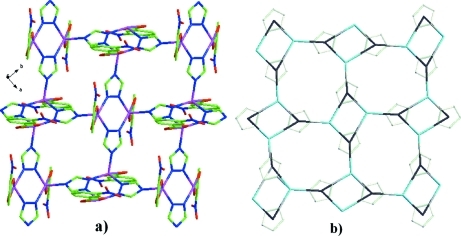
a) View of the two-dimensional net onto the ab plane, formed by cobalt ions and trz ligands; b) View of the two-dimensional net built on three-connected trz and cobalt nodes.

## supplementary crystallographic information

### Comment

Recently, more and more attention is paid on the coordination chemistry about trz ligand or analogy ligand (Park *et al.*, 2006; Yang *et al.*, 2008; Zhai *et al.*, 2007), driven by their intriguing topological matrix and potential applications.

The asymmetric unit of I is shown in Fig. 1. The Co^II^ atom is five-coordinated by two *L* (3-nitrobenzoate anion) O atoms, three trz N atoms to give rise to a distorted trigonal-bipyramidal geometry. The Co—O/N bond lengths of 2.0008 (12)–2.3314 (12)Å (Table 1) are in the normal range (Zhang *et al.*, 2008). The trz and *L* ligand adopt bridging and bidentate coordinated modes, respectively. As shown in Fig. 2a, the Co^II^ atoms are combined together by trz ligands to generate a two-dimensional net parallel to the *ab* plane with the *L* ligands ligated on the two-dimensional net up and down. From a topological point of view, if considering the trz ligands and cobalt ions as three-connected nodes. Moreover, besides the presence of two weak intermolecular C—H···O hydrogen bonds, see Table 2, there is not other obvious supramolecular interactions between two-dimensional nets,

### Experimental

CoCl_2_ (1.0 mmol), 3-nitrobenzoic acid (1 mmol) and triazole (1 mmol) were dissolved in water (10 ml). The solution was heated in a 25 ml Teflonlined reaction vessel at 433 K for *ca* 3 days and then cooled to room temperature. Purple crystals of the title compound were obtained in a yield of 78%.

### Refinement

All H atoms were positioned geometrically and refined using a riding model with C—H = 0.93 Å and with *U*_iso_(H) = 1.2*U*_eq_(C).

### Crystal data

**Table:**

[Co(C_2_H_2_N_3_)(C_7_H_4_NO_4_)]	*F*(000) = 1176
*M**_r_* = 293.11	*D*_x_ = 1.797 Mg m^−^^3^
Orthorhombic, *P**b**c**a*	Mo *K*α radiation, λ = 0.71073 Å
Hall symbol: -P 2ac 2ab	Cell parameters from 15896 reflections
*a* = 9.2419 (18) Å	θ = 3.1–27.5°
*b* = 10.377 (2) Å	µ = 1.60 mm^−^^1^
*c* = 22.597 (5) Å	*T* = 296 K
*V* = 2167.1 (8) Å^3^	Block, purple
*Z* = 8	0.14 × 0.12 × 0.12 mm

### Data collection

**Table:**

Bruker SMART 1K CCD area-detector diffractometer	2477 independent reflections
Radiation source: sealed tube	2245 reflections with *I* > 2σ(*I*)
graphite	*R*_int_ = 0.029
Detector resolution: 8.192 pixels mm^-1^	θ_max_ = 27.5°, θ_min_ = 3.1°
ω scans	*h* = −12→11
Absorption correction: multi-scan (*SADABS*; Sheldrick, 2004)	*k* = −13→12
*T*_min_ = 0.802, *T*_max_ = 0.826	*l* = −29→29
19233 measured reflections	

### Refinement

**Table:**

Refinement on *F*^2^	Primary atom site location: structure-invariant direct methods
Least-squares matrix: full	Secondary atom site location: difference Fourier map
*R*[*F*^2^ > 2σ(*F*^2^)] = 0.023	Hydrogen site location: inferred from neighbouring sites
*wR*(*F*^2^) = 0.059	H-atom parameters constrained
*S* = 1.04	*w* = 1/[σ^2^(*F*_o_^2^) + (0.0278*P*)^2^ + 1.1998*P*] where *P* = (*F*_o_^2^ + 2*F*_c_^2^)/3
2477 reflections	(Δ/σ)_max_ = 0.001
163 parameters	Δρ_max_ = 0.33 e Å^−^^3^
0 restraints	Δρ_min_ = −0.28 e Å^−^^3^

### Fractional atomic coordinates and isotropic or equivalent isotropic displacement parameters (Å^2^)

**Table:**

	*x*	*y*	*z*	*U*_iso_*/*U*_eq_	
C1	0.0918 (2)	0.62096 (17)	0.17212 (8)	0.0358 (4)	
C2	−0.0058 (2)	0.7078 (2)	0.14920 (8)	0.0470 (5)	
H2	−0.0198	0.7141	0.1085	0.056*	
C3	−0.0823 (3)	0.7853 (2)	0.18746 (9)	0.0522 (6)	
H3	−0.1473	0.8457	0.1728	0.063*	
C4	−0.0620 (2)	0.77306 (18)	0.24798 (8)	0.0406 (4)	
H4	−0.1157	0.8240	0.2738	0.049*	
C5	0.1165 (2)	0.60861 (16)	0.23241 (7)	0.0322 (4)	
H5	0.1843	0.5503	0.2468	0.039*	
C6	0.03721 (19)	0.68583 (16)	0.27043 (7)	0.0295 (3)	
C7	0.05354 (18)	0.67429 (16)	0.33614 (7)	0.0290 (3)	
C8	0.32376 (16)	0.75394 (14)	0.51070 (7)	0.0253 (3)	
H8	0.3425	0.6826	0.5344	0.030*	
C9	0.22994 (17)	0.87843 (13)	0.44808 (6)	0.0231 (3)	
H9	0.1693	0.9118	0.4190	0.028*	
Co1	0.08538 (2)	0.615646 (17)	0.442261 (8)	0.01829 (7)	
N1	0.21652 (13)	0.75890 (11)	0.47077 (5)	0.0227 (2)	
N2	0.39953 (13)	0.86141 (12)	0.51260 (5)	0.0225 (3)	
N3	0.33828 (13)	0.94272 (11)	0.47154 (5)	0.0209 (2)	
N4	0.1706 (2)	0.53740 (17)	0.13114 (7)	0.0459 (4)	
O1	−0.00457 (13)	0.75383 (12)	0.36970 (5)	0.0367 (3)	
O2	0.12664 (15)	0.58069 (12)	0.35684 (5)	0.0351 (3)	
O3	0.2746 (2)	0.47839 (18)	0.14938 (7)	0.0683 (5)	
O4	0.1257 (2)	0.53020 (16)	0.08006 (6)	0.0627 (4)	

### Atomic displacement parameters (Å^2^)

**Table:**

	*U*^11^	*U*^22^	*U*^33^	*U*^12^	*U*^13^	*U*^23^
C1	0.0517 (11)	0.0329 (9)	0.0228 (8)	−0.0083 (7)	0.0032 (7)	−0.0016 (6)
C2	0.0745 (14)	0.0453 (11)	0.0211 (8)	−0.0027 (10)	−0.0091 (8)	0.0050 (8)
C3	0.0778 (16)	0.0444 (12)	0.0345 (10)	0.0148 (10)	−0.0150 (9)	0.0058 (9)
C4	0.0592 (11)	0.0331 (9)	0.0295 (9)	0.0079 (8)	−0.0040 (8)	0.0000 (7)
C5	0.0422 (9)	0.0299 (8)	0.0246 (8)	−0.0020 (7)	−0.0011 (7)	0.0013 (6)
C6	0.0418 (9)	0.0261 (8)	0.0208 (7)	−0.0037 (7)	−0.0026 (6)	0.0022 (6)
C7	0.0371 (8)	0.0284 (8)	0.0214 (7)	−0.0058 (6)	−0.0020 (6)	0.0014 (6)
C8	0.0290 (7)	0.0196 (7)	0.0274 (7)	−0.0033 (6)	−0.0042 (6)	0.0061 (6)
C9	0.0269 (7)	0.0190 (7)	0.0234 (7)	−0.0015 (5)	−0.0031 (5)	0.0034 (5)
Co1	0.02203 (12)	0.01445 (11)	0.01838 (11)	−0.00039 (6)	0.00086 (7)	0.00145 (7)
N1	0.0263 (6)	0.0181 (6)	0.0238 (6)	−0.0032 (5)	−0.0026 (5)	0.0025 (5)
N2	0.0247 (6)	0.0185 (6)	0.0243 (6)	−0.0015 (5)	−0.0038 (5)	0.0050 (5)
N3	0.0246 (6)	0.0163 (6)	0.0217 (6)	−0.0005 (4)	−0.0008 (4)	0.0039 (5)
N4	0.0611 (11)	0.0437 (9)	0.0330 (8)	−0.0104 (8)	0.0139 (7)	−0.0058 (7)
O1	0.0453 (7)	0.0413 (7)	0.0234 (6)	0.0066 (6)	−0.0001 (5)	−0.0033 (5)
O2	0.0541 (7)	0.0304 (6)	0.0207 (5)	0.0052 (6)	−0.0027 (5)	0.0020 (5)
O3	0.0667 (11)	0.0791 (12)	0.0590 (10)	0.0140 (10)	0.0135 (8)	−0.0168 (9)
O4	0.1046 (13)	0.0585 (10)	0.0249 (7)	−0.0100 (9)	0.0117 (8)	−0.0095 (7)

### Geometric parameters (Å, °)

**Table:**

C1—C2	1.376 (3)	C8—N2	1.3175 (19)
C1—C5	1.388 (2)	C8—N1	1.3414 (19)
C1—N4	1.463 (2)	C8—H8	0.9300
C2—C3	1.376 (3)	C9—N3	1.3149 (19)
C2—H2	0.9300	C9—N1	1.3478 (18)
C3—C4	1.386 (3)	C9—H9	0.9300
C3—H3	0.9300	Co1—O1	2.3314 (12)
C4—C6	1.385 (2)	Co1—O2	2.0008 (12)
C4—H4	0.9300	Co1—N1	2.0232 (12)
C5—C6	1.385 (2)	Co1—N2^i^	2.0118 (12)
C5—H5	0.9300	Co1—N3^ii^	2.0385 (12)
C6—C7	1.497 (2)	N2—N3	1.3759 (16)
C7—O1	1.243 (2)	N4—O3	1.212 (2)
C7—O2	1.272 (2)	N4—O4	1.229 (2)
			
C2—C1—C5	122.56 (17)	N1—C9—H9	123.7
C2—C1—N4	118.45 (17)	O2—Co1—N2^i^	132.33 (5)
C5—C1—N4	118.98 (17)	O2—Co1—N1	109.04 (5)
C1—C2—C3	118.87 (17)	N2^i^—Co1—N1	105.25 (5)
C1—C2—H2	120.6	O2—Co1—N3^ii^	95.03 (5)
C3—C2—H2	120.6	N2^i^—Co1—N3^ii^	103.60 (5)
C2—C3—C4	119.79 (19)	N1—Co1—N3^ii^	109.64 (5)
C2—C3—H3	120.1	O2—Co1—O1	60.06 (5)
C4—C3—H3	120.1	N2^i^—Co1—O1	88.82 (5)
C6—C4—C3	120.72 (18)	N1—Co1—O1	89.18 (5)
C6—C4—H4	119.6	N3^ii^—Co1—O1	153.26 (5)
C3—C4—H4	119.6	C8—N1—C9	102.90 (12)
C6—C5—C1	117.93 (16)	C8—N1—Co1	128.95 (10)
C6—C5—H5	121.0	C9—N1—Co1	127.60 (10)
C1—C5—H5	121.0	C8—N2—N3	106.17 (12)
C4—C6—C5	120.10 (15)	C8—N2—Co1^iii^	124.78 (10)
C4—C6—C7	118.83 (15)	N3—N2—Co1^iii^	128.36 (9)
C5—C6—C7	121.04 (15)	C9—N3—N2	105.91 (11)
O1—C7—O2	120.80 (14)	C9—N3—Co1^iv^	125.40 (10)
O1—C7—C6	120.56 (15)	N2—N3—Co1^iv^	128.05 (9)
O2—C7—C6	118.63 (15)	O3—N4—O4	123.81 (19)
N2—C8—N1	112.47 (13)	O3—N4—C1	118.64 (17)
N2—C8—H8	123.8	O4—N4—C1	117.55 (19)
N1—C8—H8	123.8	C7—O1—Co1	82.34 (10)
N3—C9—N1	112.56 (13)	C7—O2—Co1	96.61 (10)
N3—C9—H9	123.7		
			
C5—C1—C2—C3	−0.2 (3)	C7—Co1—N1—C9	25.56 (14)
N4—C1—C2—C3	178.90 (19)	N1—C8—N2—N3	0.24 (17)
C1—C2—C3—C4	−1.2 (3)	N1—C8—N2—Co1^iii^	−170.88 (10)
C2—C3—C4—C6	1.7 (3)	N1—C9—N3—N2	−0.56 (17)
C2—C1—C5—C6	1.2 (3)	N1—C9—N3—Co1^iv^	170.84 (10)
N4—C1—C5—C6	−177.94 (16)	C8—N2—N3—C9	0.19 (16)
C3—C4—C6—C5	−0.7 (3)	Co1^iii^—N2—N3—C9	170.89 (10)
C3—C4—C6—C7	−178.94 (19)	C8—N2—N3—Co1^iv^	−170.91 (10)
C1—C5—C6—C4	−0.7 (3)	Co1^iii^—N2—N3—Co1^iv^	−0.21 (18)
C1—C5—C6—C7	177.51 (16)	C2—C1—N4—O3	166.47 (19)
C4—C6—C7—O1	−10.4 (2)	C5—C1—N4—O3	−14.4 (3)
C5—C6—C7—O1	171.35 (16)	C2—C1—N4—O4	−14.4 (3)
C4—C6—C7—O2	168.80 (16)	C5—C1—N4—O4	164.78 (17)
C5—C6—C7—O2	−9.4 (2)	O2—C7—O1—Co1	−4.03 (15)
N2—C8—N1—C9	−0.55 (17)	C6—C7—O1—Co1	175.16 (15)
N2—C8—N1—Co1	171.29 (10)	O2—Co1—O1—C7	2.54 (10)
N3—C9—N1—C8	0.68 (17)	N2^i^—Co1—O1—C7	−139.56 (10)
N3—C9—N1—Co1	−171.31 (10)	N1—Co1—O1—C7	115.17 (10)
O2—Co1—N1—C8	−113.76 (13)	N3^ii^—Co1—O1—C7	−20.77 (16)
N2^i^—Co1—N1—C8	99.90 (13)	O1—C7—O2—Co1	4.68 (18)
N3^ii^—Co1—N1—C8	−10.96 (14)	C6—C7—O2—Co1	−174.52 (13)
O1—Co1—N1—C8	−171.55 (13)	N2^i^—Co1—O2—C7	53.70 (13)
O2—Co1—N1—C9	56.19 (14)	N1—Co1—O2—C7	−79.98 (11)
N2^i^—Co1—N1—C9	−90.15 (13)	N3^ii^—Co1—O2—C7	167.23 (10)
N3^ii^—Co1—N1—C9	158.99 (12)	O1—Co1—O2—C7	−2.47 (9)
O1—Co1—N1—C9	−1.60 (13)		

Symmetry codes: (i) *x*−1/2, −*y*+3/2, −*z*+1; (ii) −*x*+1/2, *y*−1/2, *z*; (iii) *x*+1/2, −*y*+3/2, −*z*+1; (iv) −*x*+1/2, *y*+1/2, *z*.

### Hydrogen-bond geometry (Å, °)

**Table:**

*D*—H···*A*	*D*—H	H···*A*	*D*···*A*	*D*—H···*A*
C3—H3···O2^v^	0.93	2.54	3.250 (3)	134
C8—H8···O4^vi^	0.93	2.46	3.372 (2)	169

Symmetry codes: (v) −*x*, *y*+1/2, −*z*+1/2; (vi) −*x*+1/2, −*y*+1, *z*+1/2.
